# Colorectal infiltrating deep endometriosis: Laparoscopic treatment. A case report

**DOI:** 10.3389/fsurg.2022.1021944

**Published:** 2022-11-03

**Authors:** Giuseppe Di Buono, Matilde Micheli, Gaia Russo, Roberta Vella, Giuseppe Amato, Girolamo Geraci, Antonino Agrusa

**Affiliations:** Department of Surgical, Oncological and Oral Sciences, University of Palermo, Palermo, Italy

**Keywords:** colorectal endometriosis, deep infiltrating endometriosis, laparoscopic segmental colonic resection, laparoscopy, endometriosis

## Abstract

Endometriosis is a disease affecting approximately 10% of women of fertile age. A particular presentation is deep infiltrating endometriosis of the rectosigmoid colon with symptoms that can mimic an intestinal obstruction or neoplasm. We report the case of a 39-year-old woman with pelvic pain during the menstrual cycle and significant anemia who presented an ectopic endometrial tissue in correspondence of the rectum. Because of the thickness of the lesion the patient underwent a segmental laparoscopic colorectal resection with end-to-end anastomosis.

## Introduction

The term endometriosis refers to the presence in an ectopic site of normal functioning endometrial tissue. The ectopic endometrial tissue, as the normal uterine mucosa, undergoes hormonal stimulation and follows the proliferative and functional changes along the menstrual course. The main clinical symptoms of endometriosis are pelvic pain, dysmenorrhea, infertility, and diarrhea or constipation. Rarely, when endometriosis involves the gastrointestinal tract (small bowel and/or colon and/or rectum), patients can present symptoms of intestinal obstruction due to stenosis (less than 15% of the cases). Although the exact frequency of intestinal endometriosis is difficult to define because of the lack of specific symptoms, it has been estimated that implants to the bowel may occur in 3%–37% of women affected by endometriosis and the rectosigmoid junction is the most common site of localization (1). The American Society for Reproductive Medicine defined a new classification of endometriosis based on the location, amount, depth, and size of the endometrial tissue and each score was reclassified as 1–5, 6–15, 16–40, and more than 40 (2). In this report, we present a case of colorectal deep invasive endometriosis treated with laparoscopic resection in line with the CARE guidelines.

## Patient information

A 39-year-old childless Caucasian woman referred a long history of tenesmus, diffuse pelvic and abdominal pain during the menstrual cycle, dysmenorrhea, rectorrhagia, and discrete anemia (Hb 10.5 g/dl). Her medical history was negative for other diseases. Her family history was negative for neoplastic diseases.

## Clinical findings and diagnostic assessment

**Figure 1:**
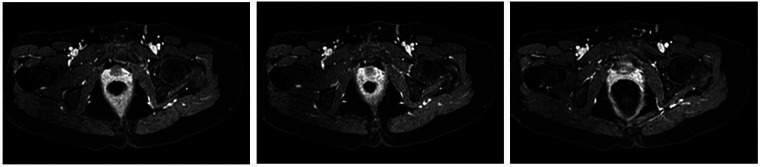
MRI illustration of the colorectal lesion with inhomogeneous signal intensity and involvement of serosa, muscular layers, and submucosa.

The patient was examined by a specialized gynecologist and a pelvic ultrasound with a diagnosis of suspected endometriosis of the left ovary was sent to our observation to determine the cause of rectal symptoms. The patient then underwent a colonoscopy, which revealed a 4 cm solid lesion occupying more than 50% of the lumen 11 cm from the anal verge. Endoscopic biopsies were carried out with histologic findings of superficial fragments of mucosa of the large intestine site of discrete lymphocytic infiltrate of the lamina propria mixed with fragments of hyperplastic polyp. There was no indication of deep endometriosis in the endoscopic biopsy. On radiological investigation, a contrast-enhanced CT scan of the chest and abdomen showed no primary or secondary lesions of the lungs, liver, or other visceral organs, as well as no abdominal-pelvic lymphadenopathy. Since the CT scan was negative, the patient underwent to pelvic MRI to better study the rectal lesion, which revealed tissue formation of the size of 2 cm × 2 cm × 2.4 cm with inhomogeneous signal intensity and endowed with discrete post contrastographic enhancement at the level of the proximal rectum and with wide extension to the wall layers but with preservation of the mucosa (Figure 1). The MRI signal characteristics of such tissue nodulation were similar to the uterine ones with radiological diagnosis of colorectal deep invasive endometriosis.

## Therapeutic intervention

**Figure 2:**
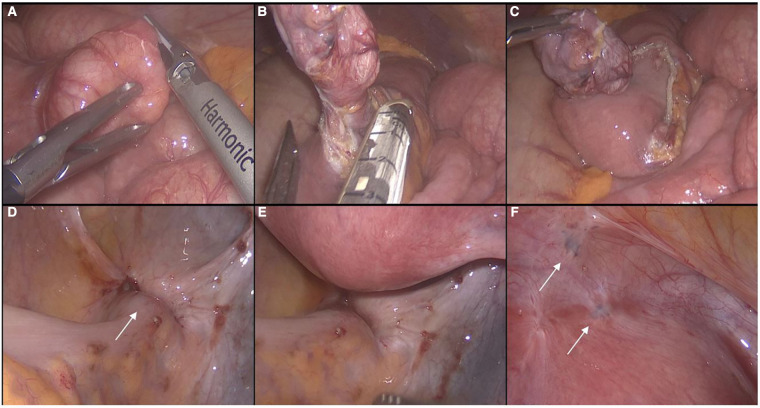
Laparoscopic exploration of the abdominal cavity; (**A**) identification of jejunal diverticulum; (**B**) isolation and excision with a linear stapler; (**C**) resected diverticulum and particular of tangential suture; (**D**,**E**) location of deep infiltrating endometriosis of the superior rectum; (**F**) other millimetric foci of endometriosis on the parietal pelvic peritoneum over the bladder-uterine cavity.

**Figure 3:**
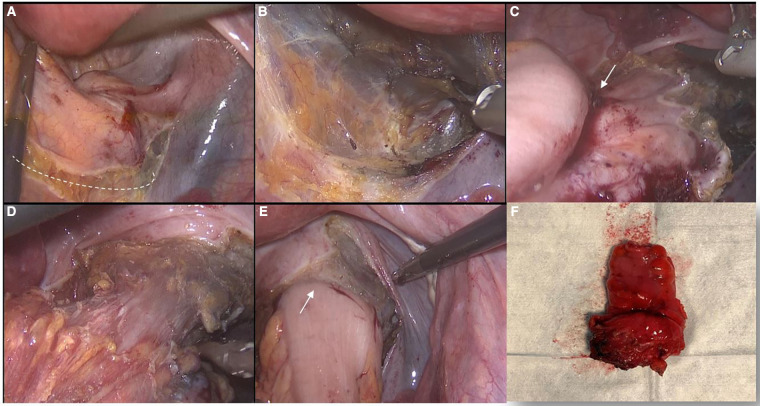
Surgical steps of laparoscopic segmental colorectal resection for endometriosis; (**A**) posterior approach to the mesorectum: the dashed line indicates the correct dissection plane; (**B**) sharp dissection of mesorectum in order to preserve the hypogastric nerves and vascular structures; (**C**) anterior dissection along Denonvilliers fascia below the endometriosis lesion; (**D**) complete surgical preparation of the rectum; (**E**) end-to-end anastomosis; (**F**) resected specimen.

Considering the age of the patient, painful symptoms with worse quality of life, refusal of medical hormonal therapies, and desire to preserve fertility, the patient was scheduled for surgery. A laparoscopic approach was chosen. The patient received antibiotic prophylaxis as per hospital protocol (cefazolin 2 g + metronidazole 500 mg). We did not mark the lesion with ink preoperatively because it was in a very precise location and was easily visible during laparoscopic exploration or, alternatively, by intraoperative endoscopic examination. In addition, preoperative marking would have led to ink diffusion into the mesorectum and pelvic peritoneum, making dissection or identification of additional endometriosis foci difficult. With the purpose of exploring the entire abdominal cavity and eventually performing surgical procedures on both the intestinal tract and the reproductive organs, the patient was placed in a Trendelemburg position with legs apart (3, 4). A Veress needle was used to induce pneumoperitoneum in the left subcostal region (5) and the trocars were positioned as in left colectomy. The optical trocar was placed in the right para-umbilical region (2 cm from the median line) (6). Three additional trocars were inserted in the right iliac fossa (12 mm), in the right hypochondrium (5 mm), and in the left flank (5 mm). On systematic exploration of the abdominal cavity and intestinal loops, a centimeter diverticulum was found on the anti-mesenteric side of the second jejunal loop to 30 cm from the ligament of Treitz (Figures 2A–C). This diverticulum was excised with a linear stapler (EndoGIA Medtronic® 45 mm, tri-staple gold charge) for extemporaneous histologic examination to evaluate the presence of ectopic mucosa and/or other location of the disease. Intraoperative histologic examination was negative for endometriosis and diagnosed a jejunal diverticulum with normal mucosa. Then, in correspondence with the proximal rectum, an area approximately 2.5 cm in diameter was found, starting from the serosa, of hard-ligneous consistency with a depressed central area and with areas of brown-black color that confirmed the preoperative MRI and endoscopic report of suspected rectal endometriosis (Figures 2D,E). Other millimetric locations of endometriosis were observed on the left parametrium in the parietal pelvic peritoneum over the bladder-uterine cavity. (Figure 2F) and on the left ovary. (Enzian score was B1C2; ASRM stage III; AAGL stage 3; EFI value: 6). Parietal pelvic peritonectomy including foci of endometriosis was performed. Endometriosis of the left ovary was treated with cyst removal. No other macroscopic lesions were evident in the right ovary and the recto-vaginal septum. In consideration of the extension in depth of colorectal endometriosis that contraindicated conservative management with disc excision it was decided to perform a segmental colonic resection (Figure 3). In consideration of the anatomy of the mesorectum and the location of the deep infiltrating endometriosis, posterior access to the mesorectum from the sacral promontory was made. For surgical dissection, we used an ultrasonic scissor with a marginal section of the vascular branches of the segment to be resected and preservation of the inferior mesenteric vessels. Then, the upper rectum, below the site of disease, was dissected with a linear stapler (EndoGIA Medtronic® 45 mm, n. 2 tri-staple purple charge). We used a suprapubic mini-laparotomy for the extraction of the surgical specimen and the final section of the sigmoid colon. An end-to-end intracorporeal trans-anal colorectal anastomosis was achieved using a circular stapler. Finally, the anastomosis was tested, and the results were negative for hydropneumatic proof.

## Follow-up and outcome

The postoperative course was regular, with no complications or indices of inflammation. The bladder catheter was removed on the first postoperative day and the patient restarted oral nutrition. The patient passed gas on POD 2 and the patient was discharged in post operative day (POD) 7. Final histologic examination confirmed the presence of connective-adipose tissue and muscle sites of endometriosis at the pelvic peritoneum and the left ovary and multiple foci of endometriosis in the submucosa, muscle tunics, and subserosa at the sigma-rectum without infiltration of the mucosa and with resection margins free of disease. At the end of the surgery, the patient did not undergo suppressive hormone therapy. After colorectal resection surgery, the patient experienced a significant improvement in abdominal symptoms with occasional pain and no rectal bleeding.

## Discussion

The clinical presentation of endometriosis (tenesmus, rectal bleeding, pelvic pain, anemia, dyspareunia) can be easily confused with other diseases, therefore the use of endoscopic investigations, with subsequent histological confirmation, and radiological studies are required to reach/establish a diagnosis. The first step of diagnostic workup when deep infiltrating endometriosis is suspected is the consultation of the multidisciplinary team made up of a gynecologist and a colorectal surgeon, who is also experienced in endometriosis surgery. One of the most important examinations for rectal endometriosis is MRI (7). The critical rectal and pararectal signs that are studied and analyzed during this radiological procedure, such as the method of assessment of circumferential extension, Sacro-recto-genital septal involvement, submucosal edema and wall thickness, may indicate whether or not segmental resection is needed. Both the clinical severity and the accurate preoperative MRI estimation aid the surgeon in the decision-making process and selection of the correct surgical treatment. In a recent study, Rousset et al. (8) compared the preoperative signs of rectal and pararectal endometriosis to establish better surgical management. In particular, the authors observed that segmental resection was required when the lesion thickness was ≥14 mm, the transversal axis was ≥22 mm, and the circumference was ≥3/8 radii (38%). Concerning pararectal involvement, only the posterior part of the Sacro-recto-genital septum was strongly associated with a significant number of colorectal resections. A multicenter retrospective study clarified that the posterolateral parametrium was a possible site of involvement together with bowel localization and had a possible functional impact with association with a higher risk of postoperative dyspareunia and sexual dysfunction (9). On the contrary, the removal of deep rectal nodules by shaving or disc excision preserves the mesorectum, rectal vasculature and nerves, as the procedure only affects the anterior rectal wall and does not change the overall length of the rectum. Mesorectal sparing is possible also in selected cases of segmental resection, especially when applying innovative intracorporeal colorectal anastomosis (10). In principle, therefore, conservative excision should have better functional outcomes than resective treatment. However, several studies compared these surgical procedures showed no significant differences in postoperative rectal function (11). In this case report, the preoperative MRI showed a lesion with a thickness of 2 cm confirmed on histologic examination with multiple foci of endometriosis until the submucosa. There were several indications for surgery, including stenosis of the colonic lumen on endoscopic exploration, the presence of severe pain symptoms, and patient preference for surgery instead of hormonal therapies. In literature, several studies considered the role of surgical treatment for deep infiltrating rectal endometriosis. The results suggested that after surgical resection, painful symptoms appear to improve by 70%, with a recurrence of symptoms from 0% to 34%. On the other hand, in a recent systematic review and meta-analysis Bafort et al. (12) identified only a randomized controlled trial by Roman et al. (13) that compared conservative laparoscopic surgery with laparoscopic colorectal resection of deep infiltrating endometriosis, revealing no significant differences in quality of life between the two techniques. Even though deep infiltrating endometriosis is a benign disease, the frequently severe clinical presentation and the intolerance to hormonal therapies could justify the surgical treatment. A multidisciplinary team approach, associated with a psychological plan for the patient, is necessary to identify the optimal management for each specific clinical presentation. In literature, there is no definitive consensus on the choice between rectal shaving, disc excision, and segmental colorectal resection as the best surgical treatment, but the use of the laparoscopic technique remains the gold standard, especially for young women in fertile age.

## Data Availability

The original contributions presented in the study are included in the article/Supplementary Material, further inquiries can be directed to the corresponding author/s.
